# Phenotypic and genotypic data integration and exploration through a web-service architecture

**DOI:** 10.1186/1471-2105-10-S12-S5

**Published:** 2009-10-15

**Authors:** Angelo Nuzzo, Alberto Riva, Riccardo Bellazzi

**Affiliations:** 1 Department of Computer Engineering and Systems Science, University of Pavia, Via Ferrata 1, 27100 Pavia, Italy; 2 Department of Molecular Genetics and Microbiology, University of Florida, Gainesville, FL, USA

## Abstract

**Background:**

Linking genotypic and phenotypic information is one of the greatest challenges of current genetics research. The definition of an Information Technology infrastructure to support this kind of studies, and in particular studies aimed at the analysis of complex traits, which require the definition of multifaceted phenotypes and the integration genotypic information to discover the most prevalent diseases, is a paradigmatic goal of Biomedical Informatics. This paper describes the use of Information Technology methods and tools to develop a system for the management, inspection and integration of phenotypic and genotypic data.

**Results:**

We present the design and architecture of the Phenotype Miner, a software system able to flexibly manage phenotypic information, and its extended functionalities to retrieve genotype information from external repositories and to relate it to phenotypic data. For this purpose we developed a module to allow customized data upload by the user and a SOAP-based communications layer to retrieve data from existing biomedical knowledge management tools. In this paper we also demonstrate the system functionality by an example application of the system in which we analyze two related genomic datasets.

**Conclusion:**

In this paper we show how a comprehensive, integrated and automated workbench for genotype and phenotype integration can facilitate and improve the hypothesis generation process underlying modern genetic studies.

## Background

One of the most challenging goals of current biomedical research is to link the genotypic and phenotypic information generated by high-throughput experimental technologies [1]. The shift from *hypothesis based *to *hypothesis-free *research that has been made possible by these technological advances opens unprecedented new opportunities for studying biological systems on a large scale, at a low cost, and with a holistic perspective that promises to expand our understanding of biological processes and of their connections with clinically relevant outcomes. The price to pay for this paradigmatic shift is that researchers will increasingly need to handle very large volumes of heterogeneous data, both generated by their own experiments and retrieved from publicly available repositories of genomic knowledge. Integration, exploration, manipulation and interpretation of such data therefore need to become as automated as possible. The "traditional" data inspection and analysis methods are quickly becoming inadequate in a scenario in which an investigator can sample hundreds of thousands of variables in parallel: not only *ad-hoc *analysis methods need to be developed in order to address problems related with the statistical significance of the analysis results, but all phases of the scientific discovery process (hypothesis generation and testing, background knowledge gathering, experiment design, interpretation of results, generation of new knowledge) will have to be adapted to this new reality. In an era in which an entire new genome can be sequenced and annotated in a matter of days, it will become essential to be able to automatically link new observations and findings to pre-existing knowledge. Finally, new data storage and retrieval systems will need to be adopted in order to handle the unprecedented volumes of data and information being generated in an efficient and productive way. These tools, however, should be easily accessible to the broad research community, facilitating the discovery process by providing high usability and effective automation. Information Technology will therefore play an increasingly crucial role in modern biomedical and translational research [2], by developing the methods and tools that will allow researchers to bridge the gap between biomedical research and clinical applications. The ability to effectively address the challenges outlined above will have a direct, dramatic impact on the speed, accuracy and effectiveness of the scientific progress in all areas of the life sciences.

We therefore propose the application of data warehouse concepts to facilitate the investigation of biomedical data by researchers lacking technical expertise and database skills [3]. Our system, called Phenotype Miner, provides a simple and effective way to organize, represent and navigate phenotypic data along multiple dimensions, and to select subsets of subjects based on one or more phenotypes of interest. Our current aim is to turn the system into a general tool for hypothesis generation, experiment design, automated annotation and biomedical data management, by relating phenotypic data to genomic knowledge. In this paper we describe the overall architecture of the system, which exploits a distributed architecture based on Web Services to integrate the Phenotype miner with two additional modules that support automated hypothesis generation process as an integral part of modern translational research. The first one is the Data Uploader, a tool to parse user-provided data sets of phenotypic data and to store them in the Phenotype Miner's database. The Data Uploader modular architecture makes it possible to easily customize both the parsing method (to support different data formats and representations) and the way in which data are stored in the database (to adapt them to the most appropriate structure for the specific analysis being performed). The second module allows the system to access external resources providing background genomic information and knowledge through a standard Web Services protocol. This tool can be used, for example, to automatically link the phenotypes under study with available genotype data on the basis of pre-existing knowledge about the relationship between the phenotypes and genomic markers.

In the following we present a detailed description of the system's architecture and usage, we illustrate the most important technological and methodological solutions we adopted for its implementation, and we present an application example. Using two sample datasets, the first one containing a dozen heterogeneous clinical measurements on about one hundred individuals and the second one containing their genotypes obtained with a SNP microarray, we demonstrate the system's usability and we highlight the ways in which it can be used to answer complex questions about genotype-phenotype correlations.

## System description

The Phenotype Miner, the core module of the system, was developed as an application of data warehousing to the domain of genetic studies. These studies rely on the integration and manipulation of large amounts of heterogeneous data, including genotypic, phenotypic and genealogical information, and therefore pose significant challenges related with structuring the data and querying them effectively. In particular, we showed how the definition and interpretation of phenotypic data can be improved through a multidimensional analysis approach [3].

### Data Warehouse techniques: the OLAP engine for data exploration

While a normalized structure (i.e, one based on the Entity-Relation model) may be preferred for a correct management of the database in terms of data integrity and reliability, the adoption of query-oriented models that reflect the logical structure of the data elements greatly facilitates the task of filtering the data on the basis of the desired combination of phenotypes and patient features. Thus, we used a logical database design technique to support end-user queries in a data warehouse called "dimensional modelling", and we adopted a data structure called "star-join schema", that has become a standard for data warehouse applications. Unlike the Entity-Relation model, a dimensional model is very asymmetric. There is one large dominant table in the center of the schema, called fact table. It is the only table in the schema which is connected to the other tables with multiple joins. Such other tables, called dimensional tables, only require a single join to be referenced by the fact table [4].

Typically a clinical database can be modeled by a star schema in which each record in the fact table represents a combination of a clinical measure and its values on a specific date for a specific patient. Therefore, the dimensions are individuals, measurement time and measurement values: all of them can be further specified using a snowflake model, that is, a model in which a given dimension has relationships to other attributes of the same dimension (used to re-normalize complex dimensions in order to eliminate redundancy) [5].

Multidimensional analysis is implemented by software tools called OLAP (Online Analytical Processing) engines. Unlike Online Transaction Processing (OLTP), where typical operations read and modify individual and small numbers of records, OLAP engines deal with large quantities of data in real time, and operations are generally read-only. The term "online" implies that even though huge quantities of data are involved – typically many millions of records, occupying several gigabytes – the system must respond to queries fast enough to allow an interactive exploration of the data.

Moreover, formalization of the phenotype definition is needed to implement automated query generation. The definition and use of a formal phenotype definition allowed us to implement an automatic query generation tool, suitable for users who may not possess the necessary technical skills in query languages and database manipulation.

**Figure 1:**
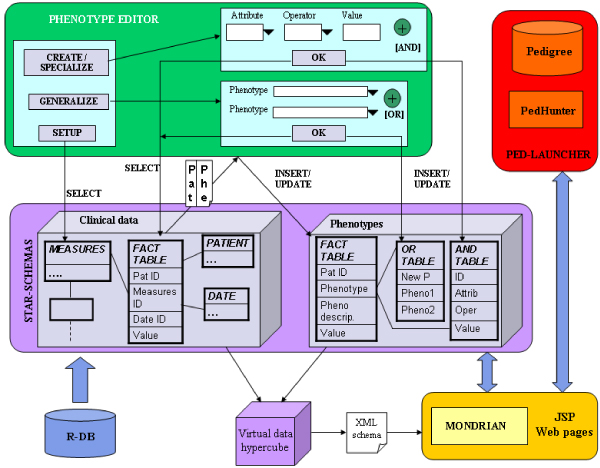
**Phenotype Miner components schemas**. An overview of the Phenotype Miner components and their interaction. The "star-schemas" section shows the data modelling schemas to perform a multidimensional analysis.

**Figure 2:**
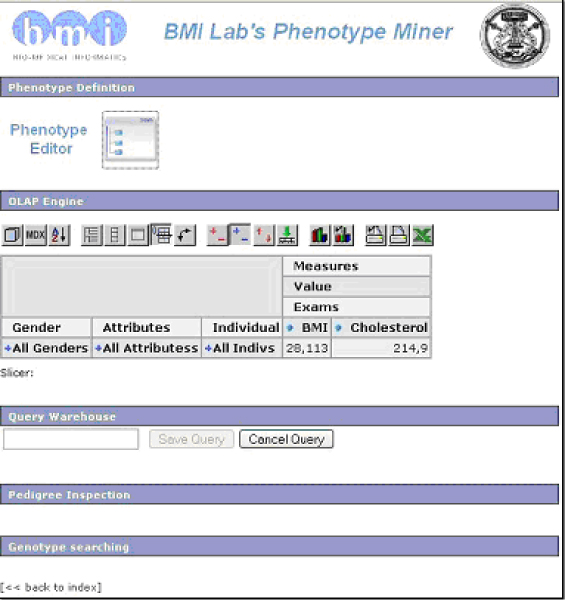
**Main Web page**. The main page of the Web interface of the system. All modules are available to the user from this main panel, and in particular the table in the OLAP engine section can be dynamically expanded by simply clicking on rows, columns and cells to examine the data according to the desired stratification strategies.

As a results, we developed the Phenotype Miner system, which included three main components: i) the Phenotype Editor, for the automated definition of phenotype queries, ii) a customized version of the Mondrian [6] OLAP engine for dynamic data inspection, iii) the PedLauncher [7] plug-in, used to map phenotype information onto the population pedigree when needed [8]. A detailed schema of the data structure and components interaction is shown and described in Figure 1, while the Web interface is presented in Figure 2.

### The Data Uploader

Phenotypic datasets are, in general, highly complex and deeply structured: the definition of the value types and ranges of the individual data elements, of the relationships that exist among them, and of the role they play is a function of the study being performed, and the structure of the database that will hold them should reflect the specific purpose for which they are going to be used. Moreover, phenotypic data comes in a very wide variety of formats, encodings and representations. It is therefore impossible to develop a single, universal method to import phenotypic data into a general-purpose tool such as the Phenotype Miner. Instead, we developed a modular component, the Data Uploader, which acts as the interface between the user-provided data source and the database. As described below, the Data Uploader can easily be reconfigured to adapt to changes in the database structure or to accept different input data formats. In this way, the Phenotype Miner can be used as an off-the shelf data mining tool, which is independent of the original data source used.

### The SOAP interface

The final component of the system is a module to communicate with external resources through a suitable Web Services interface. According to the W3C, a Web Service is defined as "a software system designed to support interoperable machine to machine interaction over a network" [9]. Web Services are Web-based communication protocols that allow a program to access one or more services available on a remote system over the network. The protocols specify how to define, locate, implement, and invoke services. In the case of SOAP [10], the most widely used protocol in the Web Services context, messages are encoded as XML documents and are transmitted between client and server using the HTTP/HTTPS protocols.

We developed a SOAP client interface by which the Phenotype Miner can access external repositories of genomic knowledge that support the Web Services protocols. In particular, we implemented methods to interact with *Genephony*, an online tool for genomic dataset annotation [11]. In the current prototype, we exploit the functionalities provided by Genephony to automatically find SNPs related to a Mendelian phenotype by searching the OMIM database [12].

Thanks to the addition of the two above-described modules, the Phenotype Miner becomes a complete, general and flexible tool to integrate phenotypic and genomic information. In the most common usage scenario, the user imports patient phenotypic data into the database using a suitable Data Uploader and uses the Phenotype Miner components (Phenotype Editor, OLAP Engine, PedLauncher) to perform the desired selection and filtering operations on the dataset. The user may then use the available external knowledge bases to integrate the phenotypic data with genomic information in order to help in their interpretation or correlate them with available data at the genomic level. Finally, the resulting dataset can be browsed or exported for subsequent analysis.

## Methods

**Figure 3:**
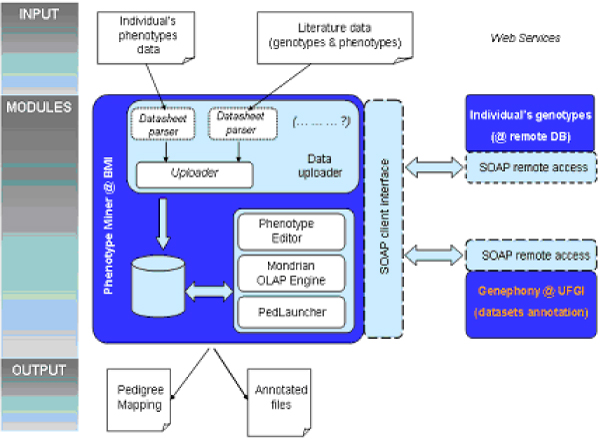
**System components overview**. An overview of the system components. The column on the left highlights the logical sections of the project.

The system was entirely developed using the Java programming language and related technologies and products. It is implemented as a servlet-based application, hosted by an Apache Tomcat Web server, and relies on a MySQL local relational database as the data warehouse. User interaction with the system takes place through a Web-based interface, and communication with external tools and resources also takes place over the Web. An overview of the system's architecture and of its components is given in Figure 3.

### The phenotype editor and the OLAP engine

Final users can create phenotype definitions using a graphical wizard developed in JAVA programming language. The tool runs as a Java Web Start application in order to exploit both the development facilities provided by stand-alone GUI APIs and to keep it available as a Web tool (instead of downloading and running it as a separate application). It interacts with two sections of the non-normalized database, i.e. the star schema of the clinical data and the phenotypes definition tables. The conditions (attributes and their values) may be defined by combo boxes, which provide lists of attributes according to the measures table of the star schema, suggesting the admissible ranges of values for each attribute. Once the rules are defined, the corresponding SQL string is created by merging conditions by AND operators, in order to: i) store the rules in the phenotype section tables, and ii) select the subgroup of individuals satisfying the conditions and storing the individuals-phenotype relationship. In the same way, it is possible to use another graphical panel to select previously defined phenotypes to be merged together by OR operators to create a new phenotype, which is then saved in the phenotypes section of the database.

When the OLAP engine starts, it reads an XML file containing the data definition. The first page of the system shows a set of check boxes containing the fields of the underlying tables, so that the user may choose the variable to be investigated (the phenotypes are among them). Once the features have been chosen, the engine loads information related to the individuals having that phenotype. Then a visual inspection of the measurement values can be performed expanding or collapsing cells of the resulting table, so that the analysis can be executed at different levels of detail.

### The Data Uploader

The Data Uploader module allows the user to upload study data from text files directly into the system's database. It is composed of different modules (called "datasheet parsers" in Figure 3), each of which is designed to parse a different type of file. The Uploader engine parses the supplied file using the appropriate datasheet parser, and inserts the resulting data into the database according to the star-schema structure. User intervention is only needed to describe the logical connections between the fields in the source files and the data elements represented in the database. We assume that the data have already been pre-processed to ensure their quality, as data cleaning and filtering are beyond the scope of this paper.

The final result is that the data are automatically stored in the data warehouse according to the star-join structure exploited by the Phenotype Miner.

It is important to note that data import is not necessarily limited to parsing local files. As the use of Web Services becomes more widespread, it is conceivable that a growing number of repositories of phenotypic data (e.g. clinical information systems) will make their data available to authorized software agents using standard interoperability protocols such as SOAP. In this case, it will be sufficient to implement a SOAP-based Data Uploader to give our system the ability to automatically acquire data from remote primary sources.

### The SOAP interface

The SOAP Interface is a module that provides the Phenotype Miner with the ability to access genomic knowledge stored within a remote resource. While there are several different types of messaging patterns available in SOAP, the most common is the Remote Procedure Call (RPC) pattern, in which one network node (the *client*) sends a request message to another node (the *server*) invoking the execution of a specified function on a specified set of arguments. The server executes the function, and automatically sends a response message back to the client with the results of the computation.

The current prototype of our system includes a SOAP interface to communicate with Genephony, an online tool for the creation and annotation of large genomic datasets. Using the services provided by Genephony, the Phenotype Miner can perform complex data annotation tasks in an efficient and straightforward way. As an example, we used the SOAP interface to retrieve the set of SNPs that are potentially related with a Mendelian phenotype of interest. This is accomplished through the following sequence of steps:

1. The client requests the creation of a *session*, in which all subsequent processing will take place;

2. The client queries Genephony for all OMIM entries containing the term (or terms) identifying the phenotype of interest; Genephony stores the resulting set in the current session;

3. The client asks for all genomic regions referenced in the OMIM entries (ie, regions known to be associated with the phenotype), and then for all SNPs belonging to these regions;

4. Finally, the client retrieves the resulting list of SNP identifiers.

The actual processing takes place on the Genephony server, with several advantages: there is no need for a local annotation database or for methods to access multiple external resources, the communication between the client and the server can be optimized (since only the final result set is transmitted to the client, and not all the intermediate steps), and the computational load on the client application is minimized. It should be noted that although OMIM only provides information about Mendelian diseases, the process just described could be applied equally well to other sources of knowledge about genotype-phenotype correlations (eg GAD, the Genetic Association Database [13]).

## Results

As an example scenario of the system usage, here we consider a case-control genome-wide association study. Association studies aim to find statistically significant differences in the distribution of a set of markers between a group of individuals showing a trait of interest (the cases) and a group of unrelated individuals who do not exhibit the trait (the controls) [14,15]. Among the several different kinds of association studies, genome-wide association studies (GWAS) rely on a set of genetic markers covering the whole genome [16]. This strategy is motivated when there is little or no *a priori *information about the location of the genetic cause of the phenotype being studied. Although the power of a genome-wide association study is usually low, such studies are useful to pinpoint areas of the genome that may contain candidate genes, and to guide a subsequent, more targeted analysis step. As the costs of genotyping decreases and the number of known, well characterized genetic markers in the human genome increases, the genome-wide approach represents an increasingly cost-effective way of generating testable research hypotheses.

Genome-wide association studies typically rely on two kinds of datasets, one collecting clinical (i.e. phenotypic) measurements, and the other one storing the individuals' genotypic markers values. In our case we assume the genotype dataset to be the result of large-scale SNP genotyping, while the clinical dataset may include any kind of measurement or observation. Starting from these two sources of information, the user's objective is to identify a set of individuals sharing a phenotype of interest, to identify a set of SNPs known to be related to the phenotype, and finally to extract from the genotype database the allelic values of the SNPs for the previously identified individuals. These steps will be reflected in the following sequence of operations in the system:

• upload the subjects' phenotype information (i.e. clinical measurements);

• define the phenotypes of interest;

• find individuals showing the defined phenotype;

• find the set of genomic markers known to be related to the phenotype;

• finally, retrieve and download genotypes for those individuals and markers only.

**Figure 4:**
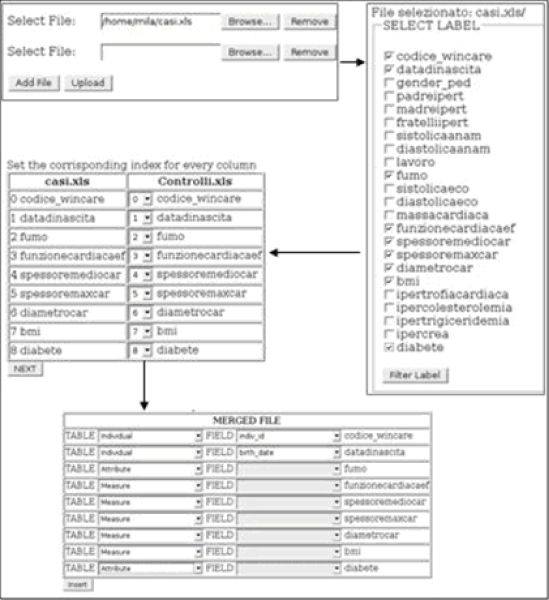
**Data Uploader**. Some of the interactive menus of the Data Uploader Web interface. After uploading one or more files, the user may specify which measurements to store in the database, and the correspondences between fields in different files, if needed.

The starting point is the file containing clinical measurements (in this example, an Excel spreadsheet). The user can upload the data into the database through the Data Uploader Web interface (Figure 4). The tool shows the contents of the datasheet in several re-organized menus, through which the user may specify additional information, such as which columns to parse, and the appropriate data type for each clinical measurement and variable. The data are then automatically inserted into the data mart on which the Phenotype Miner relies.

**Figure 5:**
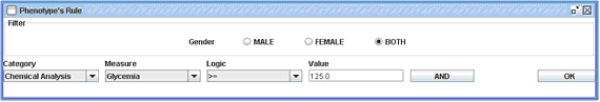
**Phenotype generation panel**. The Phenoype Editor panel for phenotype generation. Menus are automatically generated from the variables stored in the database. The rule that defines the phenotype is composed combining multiple conditions with the logical AND operator. A similar panel is also available to combine phenotypes together using the logical OR operator.

**Figure 6:**
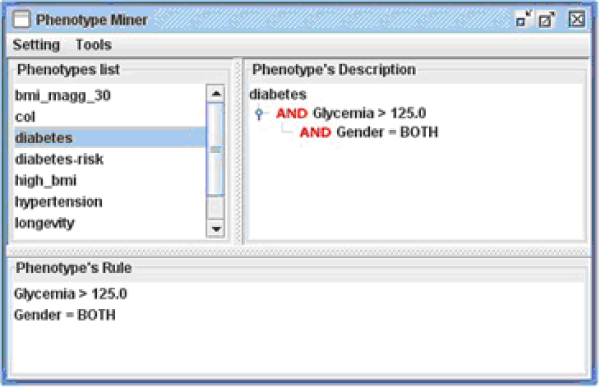
**Phenotype Editor main panel**. The main panel of the Phenotype Editor, showing phenotype definitions represented as a logical tree model. The top left frame contains the list of phenotypes defined so far, the top right frame shows the logical definition of the selected phenotype, and the bottom frame displays the same definition in textual form.

Once the data are in the system database, the user may start defining the phenotype to investigate using the Phenotype Editor. Let us suppose that the user is interested in studying individuals affected by diabetes-induced hyperglycemia. This condition can be defined as a set of logical statements on clinical measurements and other variables: for example, the *diabetes *phenotype may be defined as: "glycemia > 125 mg/dl AND gender = both" (Figure 5). Once a phenotype has been defined in this way, the system automatically generates and executes a query to identify individuals that meet the specified definition (Figure 6).

**Figure 7:**
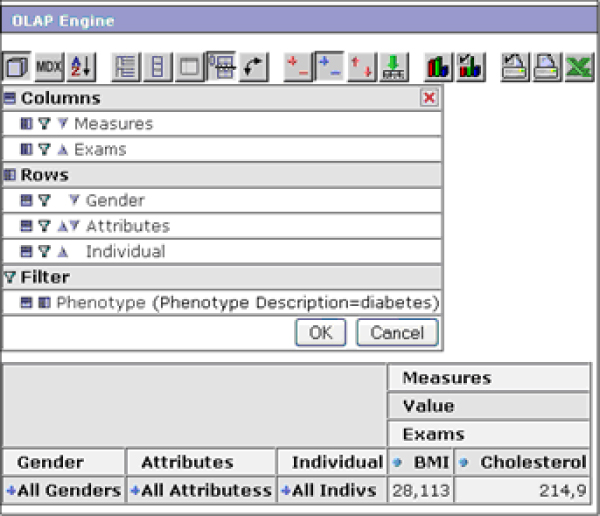
**The OLAP engine Web interface**. A detail of the OLAP engine section of the Phenotype Miner. The first button of the action bar allows the selection of the fields to be visualized in the dynamic table. The phenotypes defined by using the Phenotype Editor are listed as filters.

**Figure 8:**
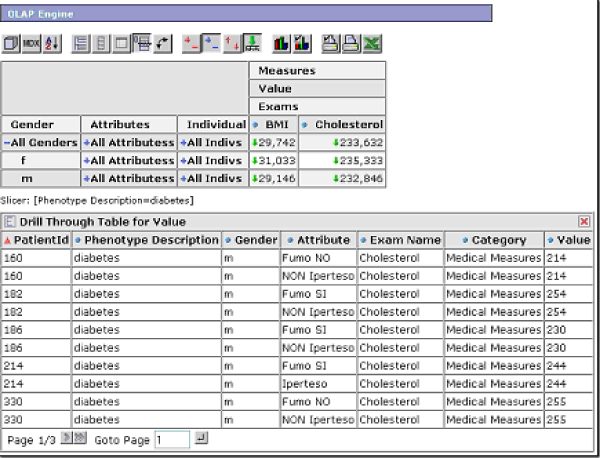
**Dill-down example**. An example of a drill-down of the table, showing detail data on the patient's phenotypes and on their clinical variables.

Internally, phenotypes are represented as *filters*, one of the functionalities provided by the OLAP engine (Figure 7). When the user selects one of them, the dynamic navigation table in the Web interface main page is automatically loaded with all the data regarding the individuals showing the selected phenotype. The user can then stratify the data at different levels of detail, simply by expanding or collapsing the table rows and columns (Figure 8).

The next step is to retrieve a set of markers that are known to be related to the phenotypes. Through the automatic SOAP request to Genephony described above, the user can retrieve all the SNPs (if any) related to the OMIM entry associated with the phenotype under study. In this example, the dataset produced by Genephony in response to the query contains about 48,000 SNPs. Of course not all SNPs will be actually needed for the purposes of the study, since many SNPs will be redundant with each other because of their proximity, and, more importantly, not all of them will be represented on the genotyping microarray used in the analysis.

Therefore, a further processing step consists in filtering the resulting set of SNPs according to the specific genotyping platform used in the study, and to other desired properties (e.g., validated SNPs only). This step too can be performed through a SOAP request to a genomic annotation service such as Genephony.

**Figure 9:**
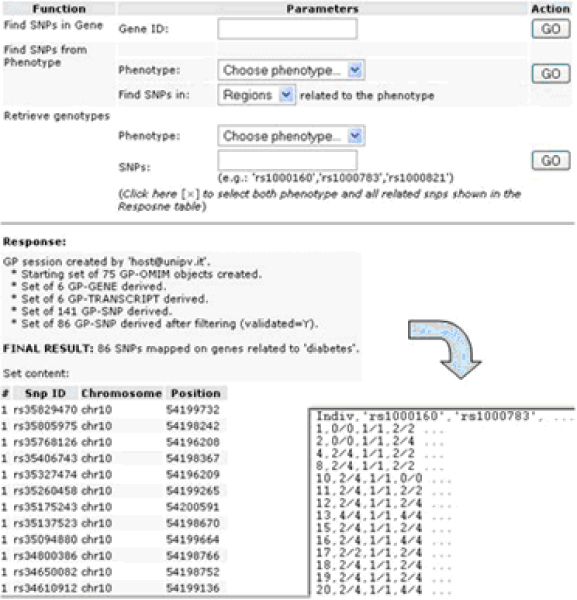
**'Genetic Inspection' section**. A summary of the Genetic Inspection section, by which SOAP-based requests are sent to remove servers, using Web Services protocols, in order to retrieve both genomic information and genotype values. The final result is a downloadable text file, which is formatted to be read by the most common genetic analysis software tools.

Finally, the system needs to retrieve the allelic values of the selected individuals only for SNPs that are known to be related to the phenotype and present in the user's database. Since the genotyping results may in general be stored in a remote database, we have used the SOAP protocol again to allow the Phenotype Miner to retrieve them. The remote database provides a SOAP service that takes as input a list of subject identifiers and a list of SNP identifiers, and returns the encoded genotypes for the specified subset of individuals and SNPs. The resulting data table is proposed to the user in a common tabular format, in which the selected individuals are listed in the rows, and the SNPs genotype values are in the columns. The table may be downloaded as a text file for further analysis through external genetic analysis software tools (Figure 9).

**Figure 10:**
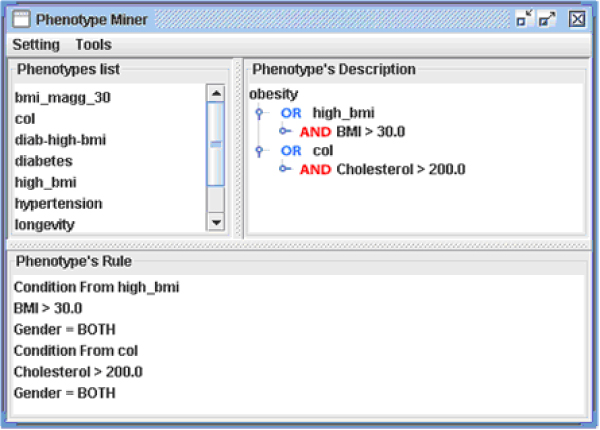
**A more complex phenotype definition**. A more complex phenotype definition, which exploits the logical tree model of the Phenotype Editor.

So far, we have shown how to retrieve specific genotypic information that is related to a phenotype of interest. Using the same sequence of operations it is possible to investigate whether genetic differences arise when including other covariates in the phenotype definition. For example, we could investigate if the SNP patterns related to the previous definition of diabetes would be different in individuals who also have an obesity problem, by including conditions on BMI or cholesterol values in the new phenotype definition (Figure 10).

Since the phenotype definition is different, the system will not only retrieve a different set of patients, but also a (possibly) different set of genetic markers. After performing association analysis in the two cases, it would therefore be possible to compare the resulting sets of associated markers directly, for example to determine if some of them are associated with both phenotypes, or to identify markers that are associated with only one of the two conditions.

Thanks to the ability to access external resources for genomic annotation, the system could then provide the user with detailed information about the genomic context of the markers thus identified (e.g. proximity to genes, biological pathways the affected genes belong to, etc). The system here described can therefore be used to generate hypothesis about candidate genetic factors potentially involved in disease development, through dynamic and powerful data exploration and annotation features.

## Conclusion

In this paper we presented a web-based software system aimed at addressing the problem of managing and exploring heterogeneous phenotypic and genotypic information in an automated, integrated and easy to use application. After describing the main system, called Phenotype Miner, which allows a dynamical inspection of clinical data, we described in more detail a set of desirable system's functionalities that we developed in order to: i) allow the user to upload experimental data, and ii) retrieve genomic information from existing biological knowledge bases and integrate it with user-supplied data. The typical application scenario of our system is in the context of studies aimed at investigating genetic factors potentially underlying phenotypes of interest.

We integrated different methodologies to develop the various software components of our system. We used a logical formalization for phenotype definition, a powerful graphical tool to define phenotypes, a data warehouse approach for dynamic, multi-dimensional data investigation, and a "Web Services" architecture to access external sources of genomic data and knowledge. The modular nature of the system makes it suitable for the development of new applications that integrate clinical data with already available external services and information resources. Future developments will concern the inclusion of new sources of information, such as investigation results from the literature, and a post-processing phase to provide specific output data formats for genetic analysis software, as well as including some preliminary statistical analysis.

A prototype version of the system is freely available at the URL . A pre-populated example database is provided for demonstration purposes.

## Competing interests

The authors declare that they have no competing interests.

## Authors' contributions

A.N. designed and implemented the Phenotype Miner system described in this paper. A.R. provided assistance with the development of the SOAP interface. R.B. contributed to the overall design of this project and supervised the Phenotype Miner project. All three authors participated in writing the present paper and approved it.
